# Larval mannitol diets increase mortality, prolong development and decrease adult body sizes in fruit flies (*Drosophila melanogaster*)

**DOI:** 10.1242/bio.047084

**Published:** 2020-01-02

**Authors:** Meghan Barrett, Katherine Fiocca, Edward A. Waddell, Cheyenne McNair, Sean O'Donnell, Daniel R. Marenda

**Affiliations:** 1 Department of Biology, Drexel University, Philadelphia, PA, USA 19104; 2 Department of Biodiversity, Earth and Environmental Science, Drexel University, Philadelphia, PA, USA 19104; 3 Department of Neurobiology and Anatomy, Drexel University College of Medicine, Philadelphia, PA, USA, 19104

**Keywords:** Nutrition, Polyol, Body size, Development

## Abstract

The ability of polyols to disrupt holometabolous insect development has not been studied and identifying compounds in food that affect insect development can further our understanding of the pathways that connect growth rate, developmental timing and body size in insects. High-sugar diets prolong development and generate smaller adult body sizes in *Drosophila melanogaster*. We tested for concentration-dependent effects on development when *D. melanogaster* larvae are fed mannitol, a polyalcohol sweetener. We also tested for amelioration of developmental effects if introduction to mannitol media is delayed past the third instar, as expected if there is a developmental sensitive-period for mannitol effects. Both male and female larvae had prolonged development and smaller adult body sizes when fed increasing concentrations of mannitol. Mannitol-induced increases in mortality were concentration dependent in 0 M to 0.8 M treatments with mortality effects beginning as early as 48 h post-hatching. Larval survival, pupariation and eclosion times were unaffected in 0.4 M mannitol treatments when larvae were first introduced to mannitol 72 h post-hatching (the beginning of the third instar); 72 h delay of 0.8 M mannitol introduction reduced the adverse mannitol effects. The developmental effects of a larval mannitol diet closely resemble those of high-sugar larval diets.

This article has an associated First Person interview with the first author of the paper.

## Abstract (precis)

**Summary:** Mannitol, a sugar polyol, decreases body size and survival and prolongs development in larval *Drosophila melanogaster*. The timing of introduction during development affects the magnitude of mannitol's impact on larvae.

## INTRODUCTION

Duration of development and adult body size are controlled by three related variables in holometabolous insects: growth rate, critical weight (the point at which the developmental period is no longer affected by resource levels), and the interval to the cessation of growth (Davidowitz and Nijhout, 2004; De Moed et al., 1999). Because size and development time are controlled by the same three parameters, a direct, positive relationship is expected and typically observed between developmental duration and adult body size (Blanckenhorn, 1998; Blueweiss et al., 2013; Nijhout et al., 2010; Roff, 2000; Thomas, 1993; Zwaan et al., 1992). However, some environmental variables can differently affect growth rate, critical weight and interval to the cessation of growth, causing neutral or even negative relationships between body size and development time (De Moed et al., 1999; Nijhout et al., 2010). High-carbohydrate larval diets, specifically sucrose and glucose, lead to delays in adult eclosion (due to delayed onset of pupation, but not prolonged pupation periods), reduced survival and lower adult body mass (Chen et al., 1996; Lihoreau et al., 2016; Matzkin et al., 2011; Musselman et al., 2011; Reis, 2016).

We asked whether larval diets, including the sugar alcohol mannitol, had developmental effects similar to high-sugar diets. We previously reported that mannitol, a non-sugar polyol carbohydrate, prolonged development when fed to *Drosophila melanogaster* larvae (Fiocca et al., 2019), and larvae fed mannitol were smaller than control larvae of the same age (Barrett and Fiocca, personal observation). Mannitol is a sugar alcohol and isomer of sorbitol. It is produced naturally as a product of fermentation and is found commonly in plants, bacteria and fungi (Jamieson et al., 2001; Lewis and Smith, 1967; Onishi and Suzuki, 1968). Mannitol is used as a low-calorie sweetener, sweetening foods without increasing blood glucose levels or insulin in humans (Saha and Racine, 2011; Yao et al., 2014). However, ingestion and breakdown of mannitol by *Tribolium castaneum* beetles increased hemolymph trehalose levels, indicating mannitol may be a nutritive source of dietary carbohydrates in some insect taxa at certain life stages (Kikuta, 2018; Takada et al., 2017).

In adult *D. melanogaster*, mannitol ingestion generated concentration-dependent, female-biased mortality (Fiocca et al., 2019). However, this effect may not carry across all developmental stages. In the only study of mannitol's effects across multiple life stages, mannitol increased mortality in sweet potato whitefly (*Bemisia tabaci*) adults while nymphs saw no lethal effect (Hu et al., 2010). Mannitol can be found in both fresh and rotting fruits (where *Drosophila* larvae often feed) due to microbial fermentation, but the impact of mannitol on *D. melanogaster* larvae has not been explored (Makinen and Soderling, 1980; Onishi and Suzuki, 1968). We hypothesized that mannitol ingestion during *D. melanogaster* development would generate phenotypes similar to those produced by high-sugar diets (Matzkin et al., 2011; Musselman et al., 2011; Reis, 2016). The ability of polyols to disrupt holometabolous development has not been studied, and identifying additional compounds that affect insect development can further our understanding of the pathways that connect growth rate, developmental timing and body size in insects.

In this study, we quantified the effect of mannitol feeding as a larva on adult body size as measured by thorax length (Bergland et al., 2008; Chechi et al., 2017; Santos et al., 1994). We assessed the effects of increasing concentrations of dietary mannitol on *D. melanogaster* larval survival, and pupariation and eclosion times. We analyzed if developmental delays were due to a delay in the onset of pupariation, and/or prolonged time in the pupal stage. We also evaluated if delaying mannitol introduction to larvae by 72 h, to approximately the early third instar (Tyler, 2000), could reduce or eliminate the decreased survival and prolonged developmental duration, as would be expected if there were a sensitive period for developmental effects of mannitol ingestion.

## RESULTS

### Effects of larval ingestion of mannitol on adult body size

**Fig. 1.**
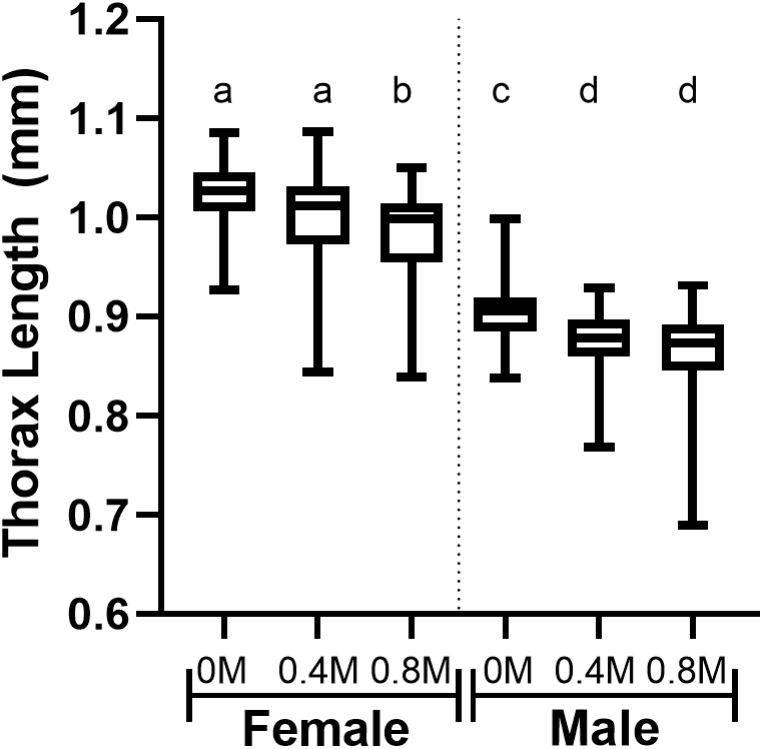
**Concentration-dependent decreases in body sizes of adult *D. melanogaster* fed mannitol as larvae.** Boxplots showing thorax lengths of males and females across increasing concentrations of mannitol; ingesting increasing mannitol concentration as larvae significantly decreases thorax lengths in emerging adults. Letters indicate significant differences between treatments (Dunn's: *P*<0.05). Linear regressions show larval ingestion of increasing mannitol concentrations decreases emerging adult thorax lengths in males and females [females: y=−0.04930x+1.022 (*F*=21.7, *P*<0.0001, R^2^=0.12; *n*=168); for males, y=−0.04644x+0.8992 (*F*=26.90, *P*<0.0001, R^2^=0.14; *n*=165)].

Adult female body size decreased as mannitol concentration increased, with 0.8 M emerging adults having smaller body sizes than 0 M or 0.4 M emerging adults (Fig. 1, Dunn's: 0–0.8 M, Z=4.44, *P*<0.0001; 0.4–0.8 M, Z=2.59, *P*=0.029; 0–0.4 M, Z=2.12, *P*=0.10). Male body size also decreased as mannitol concentration increased, with 0.8 M and 0.4 M emerging adults having smaller body sizes than 0 M emerging adults (Fig. 1, Dunn's: 0–0.8 M, Z=4.77, *P*<0.0001; 0–0.4 M, Z=4.12, *P*=0.0001; 0.4–0.8 M, Z=0.88, *P*>0.99). For females, the linear regression of mannitol concentration on body size was y=−0.04930x+1.022 (*F*=21.7, *P*<0.0001, R^2^=0.12); for males, y=−0.04644x+0.8992 (*F*=26.90, *P*<0.0001, R^2^=0.14). The slopes did not differ between males and females (*F*=0.04, *P*=0.84) indicating increasing mannitol concentration did not affect one sex's body size differently than the other (two-way ANOVA: interaction effect, *F*=1.07, d.f.=2, *P*=0.34). The intercepts were significantly different (*F*=792.6, *P*<0.0001) indicating females had larger body sizes than males at all concentrations (two-way ANOVA: sex, *F*=769.2, d.f.=1, *P*<0.0001).

### Concentration-dependent developmental delay prior to the onset of pupariation and reductions in survival

#### Developmental delay

**Fig. 2.**
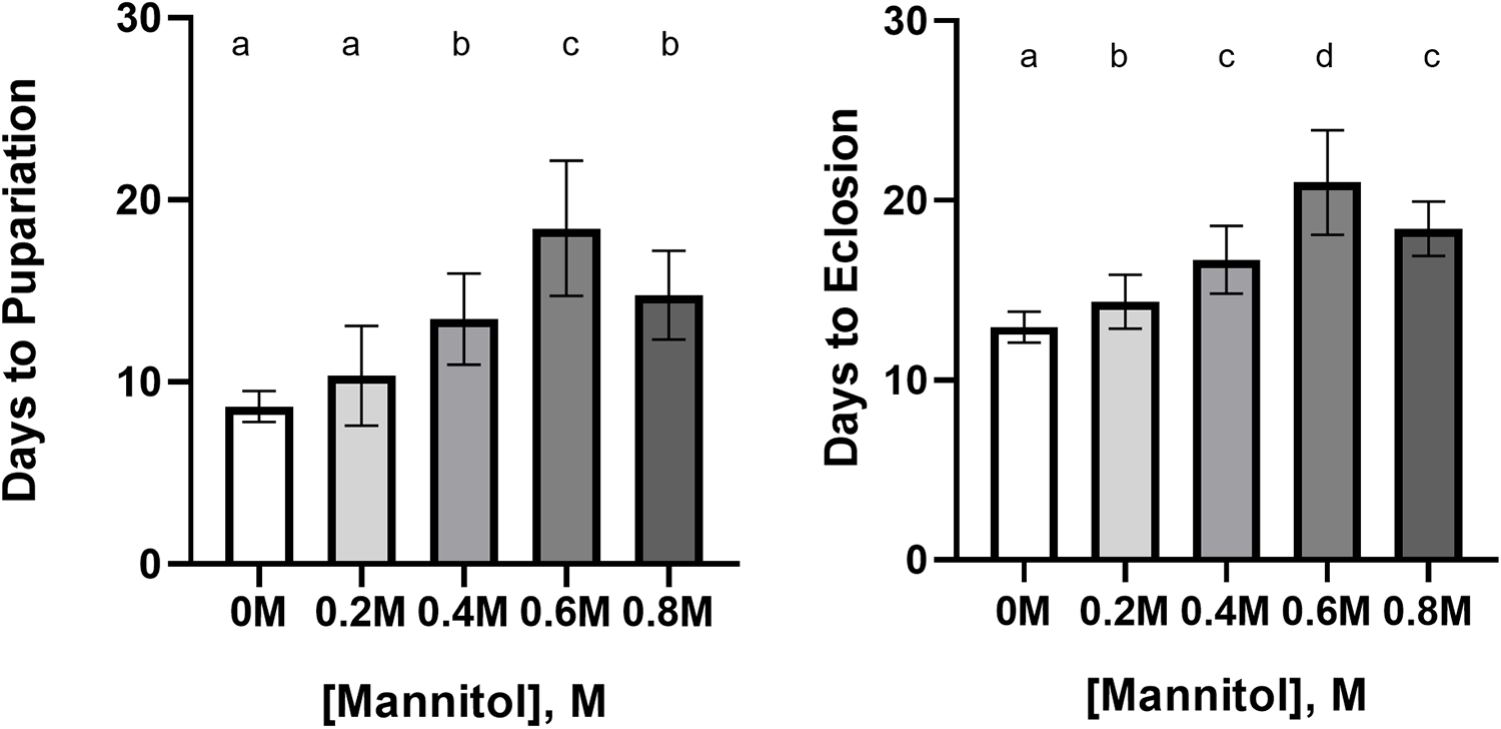
**Concentration-dependent developmental delay in *D. melanogaster* larvae fed increasing concentrations of mannitol.** (left) Time to pupariation in *D. melanogaster* larvae was significantly increased in 0.4–0.8 M conditions as compared to 0.2 M and control conditions. Letters indicate significant differences between concentrations (ANOVA with Tukey's, *P*<0.05; *n*=6 plates of five eggs/concentration). (right) Time to eclosion in *D. melanogaster* pupae was significantly increased in 0.2–0.8 M conditions. Letters indicate significant differences between concentrations (ANOVA with Tukey's, *P*<0.05; *n*=6 plates of five eggs/concentration). Error bars represent one standard deviation.

Time to pupariation was significantly increased in the 0.4 M, 0.6 M and 0.8 M conditions as compared to controls (Fig. 2, ANOVA with Tukey's: 0.4 M, q=8.61, *P*<0.0001; 0.6 M, q=14.35, *P*<0.0001; 0.8 M, q=8.97, *P*<0.0001), but not the 0.2 M condition (q=3.15, *P*=0.18). Time to adult eclosion was significantly increased in all the treatment conditions relative to controls (ANOVA with Tukey's: 0.2 M, q=4.11, *P*=0.04; 0.4 M, q=8.96, *P*<0.0001; 0.6 M, q=14.85, *P*<0.0001; 0.8 M, q=11.52, *P*<0.0001). However, the time between pupariation and eclosion was not significantly different from controls in any mannitol treatment (Fig. S2, ANOVA: *F*=1.04, *P*=0.39), indicating the major cause of eclosion delay was a delay in the onset of pupariation caused by mannitol's effects during larval development.

#### Reduced survival

We next assessed the effect of mannitol on *D. melanogaster* larval and pupal mortality. Mortality was concentration dependent for *D. melanogaster* larvae and pupae when assessed prior to eclosion, with 0.4 M, 0.6 M and 0.8 M treatments showing a significant difference from the control (Fig. S3, Mantel-Cox: 0.2 M, x^2^=0.28, *P*=0.60; 0.4 M, x^2^=9.40, *P*=0.002; 0.6 M, x^2^=23.53, *P*<0.001; 0.8 M, x^2^=19.41, *P*<0.001).

**Fig. 3.**
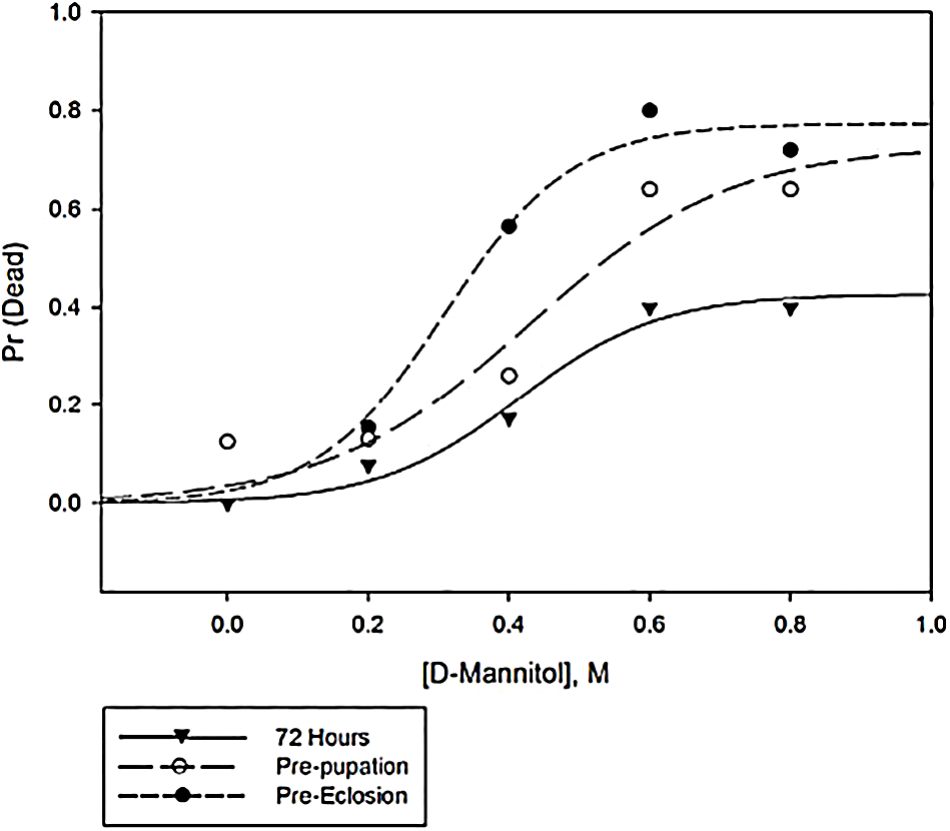
**Proportion of larvae dead after mannitol ingestion at different time points during development.** Proportion of *D. melanogaster* larvae dead at 72 h after laying eggs, prior to pupariation (inclusive of deaths at 72 h), and prior to eclosion (inclusive of 72 h and prior to pupariation deaths), across increasing concentrations of mannitol. The three-parameter best-fit sigmoidal functions are shown, and the function for pre-eclosion mortality was used to calculate the LC_50_ for *D. melanogaster* prior to eclosion (Eqn 1: 0.36 M mannitol; *n*=6 plates of five eggs/concentration).

Highly significant differences in larval mortality occurred as early as 48 h after the eggs were laid in the 0.6 M and 0.8 M (Fig. 3, Mantel-Cox: 0.6 M, x^2^=5.24, *P*=0.022; 0.8 M, x^2^=10.39, *P*=0.001) and 72 h after the eggs were laid in the 0.4 M, 0.6 M and 0.8 M (Mantel-Cox: 0.4 M, x^2^=4.47, *P*=0.035; 0.6 M, x^2^=11.81, *P*=0.001; 0.8 M, x^2^=11.88, *P*=0.001).

The best-fit sigmoidal curve for pre-eclosion LC_50_ data was:(1)
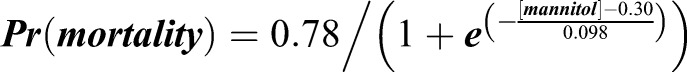
This curve was a significant fit to the data (Fig. 3; R^2^=0.96, *P*=0.039) and using the equation we found the pre-eclosion LC_50_ to be 0.36 M mannitol. Between pupariation and eclosion, 0.4 M and 0.6 M mannitol-fed flies had higher mortality compared to both 0 M (Fisher's: 0.4 M, *P*=0.0015; 0.6 M, *P*=0.0046) and 0.2 M (0.4 M, *P*=0.0023; 0.6 M, *P*=0.0062); 0 M and 0.2 M were not different from one another (*P*>0.99). The 0.8 M treatments were not significantly different from controls, but this may be an effect of small sample size due to relatively low survival through the larval stage (*n*=9 surviving pupae, Fisher's: 0 M, *P*=0.08; 0.2 M, *P*=0.09).

### Concentration-dependent reduction of mannitol's developmental effects by delaying mannitol introduction to larvae for 72 hours

#### Partial rescue of developmental delays

**Fig. 4.**
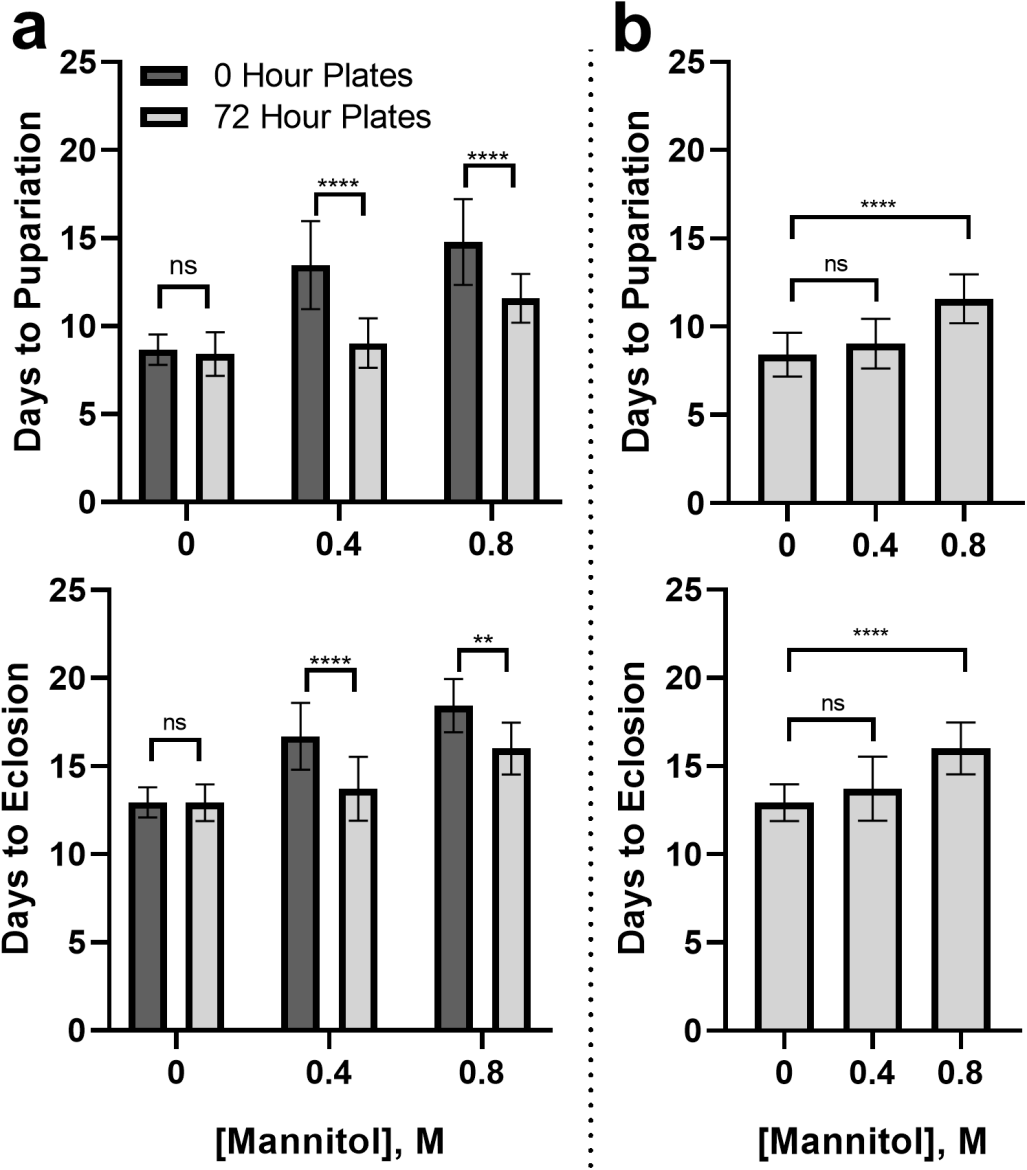
**Differences in developmental delay when mannitol introduction is postponed to 72 h.** (A) Pupariation and eclosion times in *D. melanogaster* larvae were significantly decreased in 0.4 M and 0.8 M conditions when larvae were first placed on mannitol 72 h after the eggs were laid. Asterisks indicate significant differences between 0 h and 72-h plates (Tukey's, ns, not significant; **P<0.01, ***P<0.001; *n*=6 plates of five eggs or larvae/treatment). Error bars represent one standard deviation. (B) Pupariation and eclosion times were not significantly different between 0 M and 0.4 M treatments when larvae were first placed on mannitol 72 h after the eggs were laid; larvae fed 0.8 M mannitol after 72 h still had prolonged pupariation and eclosion times. Asterisks indicate significant differences between 0 h and 72-h plates (Tukey's, ns, not significant; ****P<0.0001; *n*=6 plates of five eggs or larvae/treatment). Error bars represent one standard deviation.

Delaying introduction of mannitol to the larval diet by 72 h (72-h plates) significantly decreased pupariation and eclosion times in the 0.4 M treatment (Fig. 4A; ANOVA with Tukey's, pupariation, q=12.71, *P*<0.0001; eclosion time, q=7.94, *P*<0.0001), and the 0.8 M treatment (pupariation time: q=7.02, *P*<0.0001; eclosion time: q=5.23, *P*=0.0047) relative to plates where larvae were fed the same concentration of mannitol from hour 0 after the eggs were laid.

Pupariation and eclosion times were not significantly different from 0 M conditions in the 0.4 M 72-h plates (Fig. 4B; ANOVA with Tukey's, pupariation, q=2.00, *P*=0.72; eclosion, q=2.82, *P*=0.35). Pupariation and eclosion times were still significantly longer than controls in 0.8 M 72-h plates (pupariation: q=9.30, *P*<0.0001; eclosion: q=9.20, *P*<0.0001).

#### Partial rescue of larval survival

**Fig. 5.**
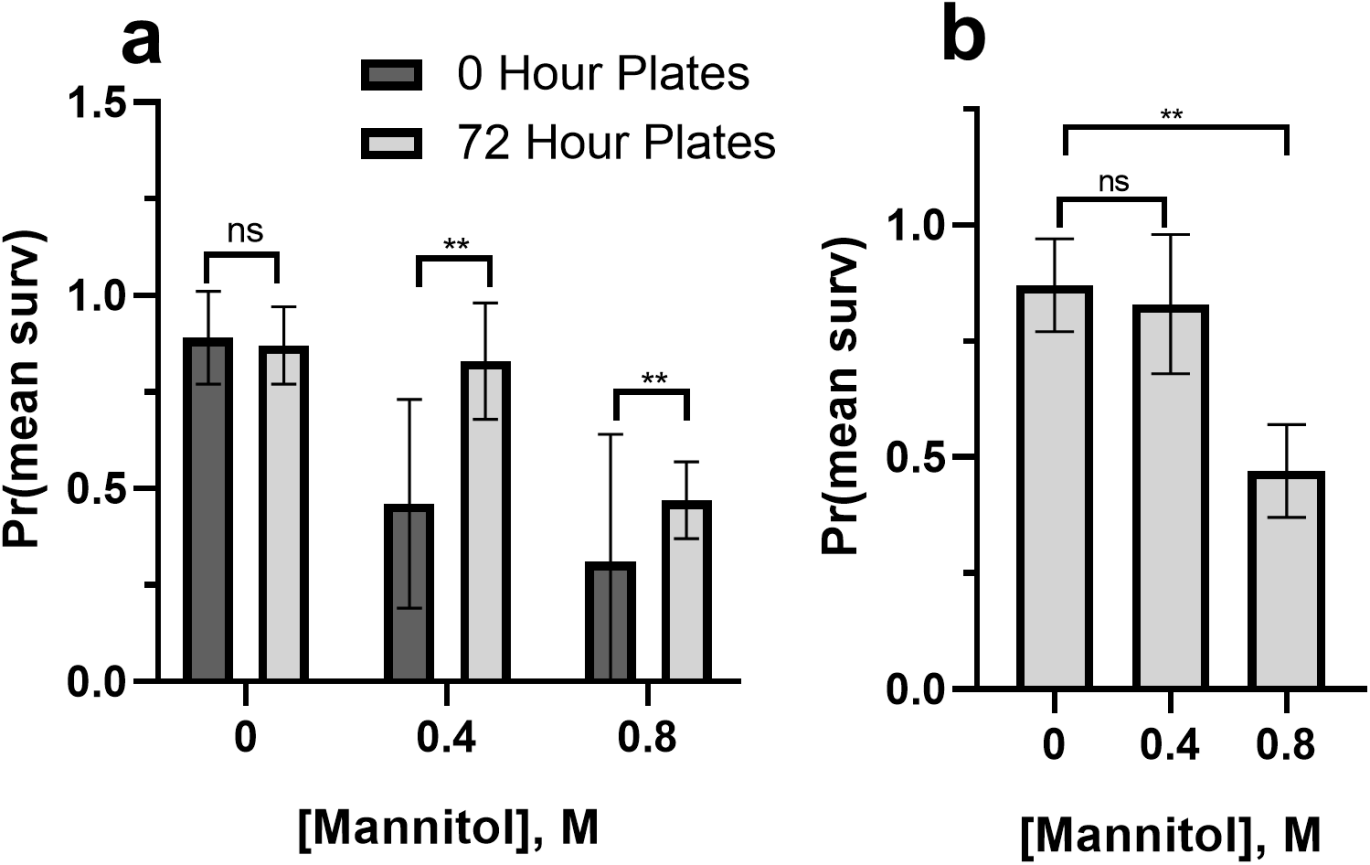
**Concentration-dependent partial rescue of survival when mannitol is introduced after 72 h.** (A) Pre-eclosion survival was significantly increased in 0.4 M and 0.8 M conditions when larvae were first placed on mannitol media after 72 h instead of at hour 0 (after the eggs were laid). Asterisks indicate significant differences between control and 72-h treatments (Mantel-Cox, ns, not significant; **P<0.01; *n*=6 plates of five eggs or larvae/treatment). Error bars represent one standard deviation. (B) When mannitol introduction to *D. melanogaster* larvae is delayed by 72 h, 0.4 M and 0 M treatments no longer differ in their survival, while 0.8 M treatments still have significantly decreased survival compared to controls. Asterisks indicate significant differences between 0 h and 72-h plates (Mantel-Cox, ns, not significant; **P<0.01; *n*=6 plates of five eggs or larvae/treatment). Error bars represent one standard deviation.

Waiting 72 h before introducing larvae to mannitol media also significantly increased survival to eclosion (relative to initiating mannitol feeding at day 0) at 0.4 M and 0.8 M mannitol concentrations (Fig. 5A, Mantel-Cox: 0.4 M, x^2^=8.91, *P*=0.003; 0.8 M, x^2^=6.80, *P*=0.009). In the 0.4 M 72-h plates, survival was not significantly different from 0 M treatment (Fig. 5B; x^2^=0.00, *P*=0.986), while the 0.8 M 72-h plates treatments were significantly different from 0 M (x^2^=8.03, *P*=0.005).

**Fig. 6.**
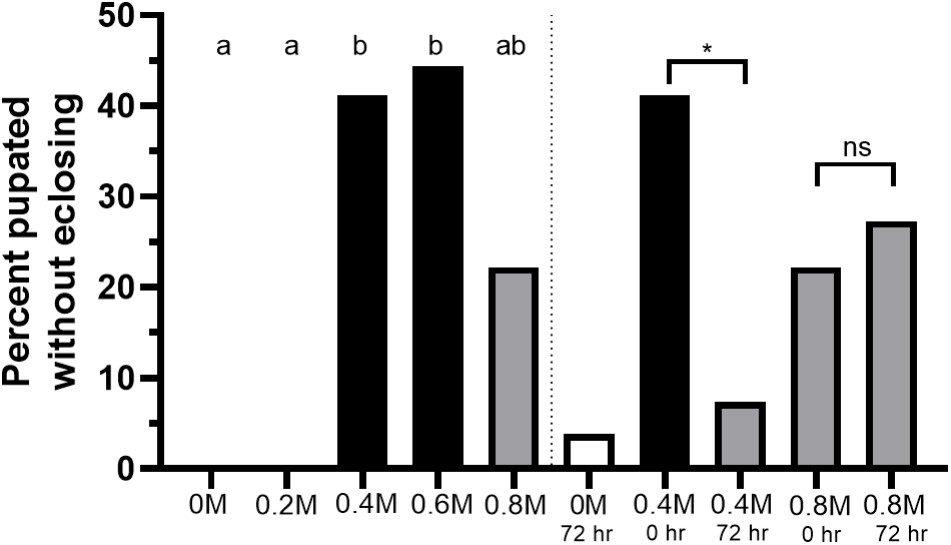
**Concentration-dependent eclosion failure, and change in eclosion failure due to delayed mannitol introduction, across increasing concentrations of mannitol.** Percent of larvae that pupated but failed to eclose across increasing concentrations of mannitol (0–0.8 M) and in 0.4 M and 0.8 M 72-h plate treatments. Letters indicate highly statistically significant differences between treatments (Fisher's: *P*<0.01; *n*=30 eggs or larvae/treatment); white=a, grey=ab, black=b. Asterisks indicate significant differences between 72 h and 0 h plates of the same concentration (Fisher's: ns, not significant; *P<0.05).

The percent of pupae that did not eclose significantly decreased in 0.4 M treatments when mannitol introduction was delayed by 72 h, but no significant difference was found between 0 h and 72 h mannitol introduction in 0.8 M treatments (Fig. 6, Fisher's: 0.4 M, *P*=0.017; 0.8 M, *P*>0.99). The percent of pupae that did not eclose in 0.4 M 72-h plates was not significantly different from 0 M controls (Fisher's: *P*>0.99).

## DISCUSSION

The typical positive relationship between holometabolous insect body size and developmental duration can be reversed under some dietary conditions (Danielsen et al., 2013; Huang et al., 2018; Reis, 2016; Xiao et al., 2005). In this study, we asked whether larval fruit flies fed mannitol diets showed developmental effects similar to those induced by high-sugar diets. We tested the effects of a mannitol diet on several aspects of larval phenotypes in *D. melanogaster*. Mannitol increased *D. melanogaster* developmental duration and decreased emerging adult body size in a concentration-dependent manner. While larval density was not controlled in our experiment for body size, increased density typically leads to decreased thorax lengths; in our least dense vials (0.8 M) we saw the smallest body sizes, indicating that larval density was not responsible for this trend (Santos et al., 1994). The phenotypic effects of a mannitol diet on the duration-size relationship in *D. melanogaster* were similar to the effects of high-sugar diets (Matzkin et al., 2011; Musselman et al., 2011; Reis, 2016; Rovenko et al., 2015).

Increased developmental duration due to mannitol ingestion was a result of delayed onset of pupariation, not prolonged pupal metamorphosis. Both the stage of larval development when mannitol was introduced (first or third larval instar), and the mannitol concentration in food, influenced the severity of mannitol's phenotypic effects. Delaying 0.4 M mannitol introduction for 72 h eliminated the effects on development time and survival; 0.8 M mannitol still had significant, although lessened, effects when introduction was delayed for 72 h. These phenotypic effects are consistent with those of high-sugar diets that generate smaller adult body sizes and prolonged development prior to the onset of pupariation (Matzkin et al., 2011; Musselman et al., 2011; Reis, 2016); as with our experiments on mannitol, the high-concentration sugar diets had stronger effects earlier in larval development (prior to the third instar) (Musselman et al., 2011).

Models of the independent effects of growth rate, critical weight, and the interval to the cessation of growth on the duration-size relationship in *Manduca sexta* indicate that variation in growth rate can lead to a negative relationship, while variations in interval to the cessation of growth and critical weight generally lead to positive relationships (Davidowitz et al., 2005; Nijhout et al., 2010). Critical weight typically occurs directly after the second molt in *D. melanogaster*, approximately 72 h post-hatching (De Moed et al., 1999; Tyler, 2000). 72 h delays in introducing high-concentration dietary mannitol still led to increased *D. melanogaster* development times, it is thus unlikely that mannitol decouples the positive duration-size relationship via altering critical weight. Instead, mannitol may impact growth rate and/or the interval to cessation of growth, potentially by disrupting the insulin/TOR signaling pathway that regulates these variables in *D. melanogaster* (Mirth and Shingleton, 2012).

In *D. melanogaster*, a carbohydrate-rich diet led to delays in eclosion and smaller pupal case sizes (Reis, 2016). Extremely high-sugar (e.g. 1 M sucrose) diets produced insulin resistance, leading to smaller wandering third instar larvae and adults irrespective of protein availability, the sugar used, or osmolarity of the food medium during development (Musselman et al., 2011). In addition, high-sugar feeding led to dramatic delays in pupariation (Musselman et al., 2011; Rovenko et al., 2015), similar to what we saw in our 0.4–0.8 M mannitol treatments. Delays in eclosion, due to high-sugar diets affecting the insulin-signaling pathway, caused delayed onset of pupariation but not prolonged metamorphosis (Matzkin et al., 2011); again, this is the same phenotype we induced when larvae were fed mannitol diets.

Because concentrations of all non-mannitol carbohydrates were kept the same in larval foods, *D. melanogaster* would need to be able to metabolize mannitol in order for it to increase levels of trehalose in the hemolymph like metabolizable sugars (glucose and sucrose). No studies have examined if *D. melanogaster,* or its common gut microbes, can metabolize mannitol, but female red flour beetles (*Tribolium castaneum*) have higher trehalose levels in the hemolymph after feeding on mannitol (Kikuta, 2018). Circulating trehalose is responsible for TOR activation in *D. melanogaster* fat bodies, contributing to cell growth during development; mannitol's catalysis to trehalose may be responsible for mediating its effects via the insulin/TOR signaling pathway, similar to other carbohydrates (Kim and Neufeld, 2015; Morris et al., 2012)*.*

Proper growth during development can also influence survival to, and in, adulthood (Mirth and Riddiford, 2007). High-sugar diets cause mortality in both *D. melanogaster* and *Drosophila mojavensis* (Matzkin et al., 2011; Rovenko et al., 2015). We found that mannitol causes mortality in *D. melanogaster* larvae after 48 h in a concentration-dependent manner, with an LC_50_ of 0.36 M. In addition, of the larvae that pupated in the 0.4 M and 0.6 M treatments, a significant number of them failed to eclose.

Starvation is a potential mechanism for mannitol's effects on larval survival; however, this is unlikely due to mismatches between starvation phenotypes and our results. Post-critical weight starvation causes accelerated emergence (our 72 h plates saw normal or delayed emergence) while pre-critical weight starvation causes developmental delay but normal adult body sizes (unlike our smaller adults) (Beadle et al., 1938). Simply reducing nutritional availability throughout development generates smaller adult body sizes, but no change in survival through eclosion (Ormerod et al., 2017). Additionally, all mannitol-fed larvae received the same basic nutrients as were fed to the controls. Mannitol may also be acting as an osmotic stressor to larvae, despite their excellent osmoregulatory ability, as mannitol is known for its diuretic effects (Croghan and Lockwood, 1959; Nicolson, 1994; Diaz-Fleischer et al., 2019; Elhassan and Schrier, 2015; Grembecka, 2015). Other species exhibit longer development times, decreased body size, reduced feeding, and/or reduced survival in osmotically stressful environments (Chinathamby et al., 2006; Clark et al., 2004; Matthews, 1985; Wu et al., 2012; Niewalda et al., 2008; Rovenko et al., 2015), which matches some of our results.

A single genetically variable insulin signaling pathway regulates growth, reproduction, longevity and metabolism in all insects, and contains conserved elements across all animals (Das and Arur, 2017; Wu and Brown, 2006). This pathway is involved in numerous environmentally-driven polyphenisms generated during development, including caste differentiation in social insects, as well as geographically- and nutritionally-driven morphological variation (De Jong and Bochdanovits, 2003; Lin et al., 2016; Lu and Pietrantonio, 2011; Wheeler et al., 2006; Xu et al., 2015). Mannitol's developmental effects provide an opportunity to compare phenotypic variation in response to the nutritional environment, generated by the evolution of insulin signaling genes across species. This study joins a growing body of work indicating that the frequently-cited positive relationship between duration of development and body size in insects can be altered by environmental variation, particularly via dietary influences. Our work also suggests that the importance of this variation, and its influence on specific developmental parameters, may change as development progresses past various sensitive periods. Mannitol's effects on development provide a novel paradigm for exploring the environmentally-cued regulation of developmental-physiological relationships in insects.

## MATERIALS AND METHODS

### Culturing *Drosophila*

Wild-type (Canton S) *D. melanogaster* (Bloomington *Drosophila* Stock Center) were raised to adulthood on standard *Drosophila* media for laboratory culturing and reared in an insect growth chamber at 27.5°C, 50% relative humidity, with a 12-h:12-h photoperiod (Chakraborty et al., 2011). These conditions were used to rear adults and for all larval experiments. Standard media was prepared in 100 ml batches as follows: 9.4 g cornmeal, 3.77 g yeast, 0.71 g agar, 0.746 ml Propionic acid, 1.884 ml Tegosept (10% w/v methyl p-hydroxybenzoate in 95% ethanol), and 9.42 ml molasses (Genesee Scientific). The appropriate amount of mannitol (HiMedia; GRM024-500G, Lot 000249743) was added, and beakers were filled with distilled water to a final volume of 100 ml. After heating the mixed ingredients to set the agar, media was poured into vials and cooled until consistency was firm and uniform. An excess of media was provided, with 10 ml in each vial.

### Testing effect of larval mannitol feeding on adult body size

Groups of 15 male and 15 female wild-type flies raised on standard media were placed in vials containing 0 M, 0.4 M, or 0.8 M mannitol adult media (standard media recipe with no molasses) and allowed to lay for 24 h (at which time they were removed). Nine vials were used per concentration, with a total of 405 flies of each sex. Vials were checked for newly emerged adults every 12 h from day 10 to day 15, and every 24 h from day 15 to day 24 (the last day that a larva pupariated in the larval plate trials). Adult flies were removed from the vials and sexed; two males and two females were randomly selected every 24 h from each vial with adults. Selected adults were euthanized and photographed for body size measurements (0M: *n*=52 females, *n*=56 males; 0.4M: *n*=66 females, *n*=61 males; 0.8M: *n*=50 females, *n*=49 males). Photographs of the thorax were taken from a dorsal view at 4 X magnification using a digital camera mounted (0.7 X) on a dissecting scope. Measurements of thorax length were taken from the tip of the scutellum to the most anterior part of the mesothorax (Bergland et al., 2008; Chechi et al., 2017) in ImageJ using the ruler tool (Schneider et al., 2012), and photographs of a stage micrometer were used to convert pixels to mm.

### Testing effects of dietary mannitol on larval mortality and developmental delay

Translucent media was produced by omitting the cornmeal from the standard media recipe and lowering the amount of agar to 0.52 g/100 ml (O'Donnell et al., 2017). Food was poured to a depth of 3 mm in 50 mm diameter petri dishes, allowing for the observation of the larvae in the food. Groups of over 100 mixed male and female wild-type flies raised on standard media were placed in each of ten egg laying chambers. At the end of 4 h, eggs were collected and five eggs were plated per petri dish, with mannitol concentrations from 0–0.8 M, at 0.2 M increments. Six petri dishes were used per concentration (*n*=30 eggs/concentration). Egg hatching, mortality, pupariation and eclosion were assessed every 24 h for 27 days using the methods detailed in (O'Donnell et al., 2017). Mean pr (mortality), days to pupariation, and days to eclosion were calculated for each concentration and a three-parameter sigmoid curve was fitted to the data to assess LC_50_ prior to eclosion.

### Testing for a change in severity of mannitol's developmental effects when delaying introduction to larvae by 72 h

Groups of approximately 100 mixed male and female wild-type flies raised on standard media were placed in each of ten egg laying chambers. At the end of 4 h, eggs were collected and plated on 0 M control translucent media where they were raised for 72 h. After 72 h, five larvae were plated per treatment petri dish (using translucent media), with the mannitol concentrations from 0–0.8 M, at 0.4 M increments. Six petri dishes were used per concentration (*n*=30 eggs/concentration). Larval mortality, pupariation, and eclosion were assessed every 24 h for another 22 days. Mean percent mortality, days to pupariation and days to eclosion were calculated for each concentration.

### Statistical analyses

Analyses were performed using SPSS v. 24, Sigmaplot v 12.5, and Graphpad v. 8.0.0 (Graphpad Prism for Windows, 2018; IBM SPSS Statistics for Windows, 2016; Sigmaplot for Windows, 2013). The effects of mannitol introduction to larvae on adult body size were analyzed using Kruskal–Wallis test with Dunn's multiple corrections for each sex. A two-way ANOVA was used to look for an interaction effect between sex and mannitol concentration on body size. A linear regression was fitted to the data for each sex across concentrations, and the slopes and intercepts were compared in Graphpad to assess if sexes differed in body size and in the degree of mannitol's effect on their body size.

Effects of eclosion day on male or female body size within a concentration were assessed using linear regressions in GraphPad, to understand the effects of mannitol in individuals that are more or less delayed in their development within a concentration and sex. This allowed us to look for any effect of day-based sampling bias, as we did not measure every emerging adult's body size, but only two per day of each sex in each vial. There was no significant trend within each pair of concentration and sex (e.g. 0 M+females) of emergence day on body size, except in 0.4 M males, indicating that flies emerging earlier and later within a concentration were not differently affected by mannitol and reducing the likelihood of day-based sampling bias on our results (Fig. S1; 0 M-female, *F*=0.47, *P*=0.50; 0 M-male, *F*=3.52, *P*=0.07; 0.4 M-f, *F*=0.80, *P*=0.37; 0.4 M-m, *F*=10.51, *P*=0.002; 0.8 M-f, *F*=0.16, *P*=0.69; 0.8 M-m, *F*= 2.00, *P*=0.16). The slopes of the regressions across all six concentration-sex pairs were not significantly different from one another (*F*=0.53, *P*=0.75).

Larval mortality data across mannitol concentrations at 48 h, 72 h and pre-eclosion was assessed using survival analyses in SPSS (Bewick et al., 2004), with subjects living to the end of the trial or eclosed included in the analysis as right-censored values on the final day of that test (48 h, 72 h and the last day of the trial, respectively). Pupae that had not eclosed after at least 6 days at the end of the trial were marked as ‘dead’ on the final day of the trial (day 27). Differences in survival distributions across concentrations were tested using pairwise log-rank Mantel Cox tests. Three-parameter, best-fit sigmoidal function LC_50_ curves for larvae at 72 h, pre-pupariation, and pre-eclosion were generated in Sigmaplot. To analyze any effects on survival of delaying the introduction of mannitol to larvae by 72 h, we used a pairwise log-rank Mantel Cox test (with subjects eclosed before the end of the trial included as right-censored values on day 25, and pupae that had not eclosed marked as ‘dead’ on the final day).

To analyze developmental delays across concentrations, we used a one-way ANOVA with Tukey's multiple comparisons test in Graphpad. To analyze differences in time from pupariation to eclosion, a one-way ANOVA with Tukey's multiple comparisons was used. To analyze any phenotypic effects on pupariation/eclosion time across replicates (*n*=6/concentration) by delaying the introduction of mannitol to larvae by 72 h, we used a two-way ANOVA and Tukey's multiple comparisons Tests in Graphpad. Differences in the number of larvae that pupated, but did not eclose, across concentrations in the delayed-introduction treatments were analyzed using Fisher's exact tests in Graphpad.

## Supplementary Material

Supplementary information

## References

[BIO047084C1] Beadle G. W., Tatum E. L. and Clancy C. W. (1938). Food level in relation to rate of development and eye pigmentation in *Drosophila melanogaster*. *Biol. Bull.* 75, 447-462. 10.2307/1537573

[BIO047084C2] Bergland A. O., Genissel A., Nuzhdin S. V. and Tatar M. (2008). Quantitative trait loci affecting phenotypic plasticity and the allometric relationship of ovariole number and thorax length in *Drosophila melanogaster*. *Genetics* 180, 567-582. 10.1534/genetics.108.088906 18716336 PMC2535706

[BIO047084C3] Bewick V., Cheek L. and Ball J. (2004). Statistics review 12: survival analysis. *Crit. Care* 8, 389-394. 10.1186/cc2955 15469602 PMC1065034

[BIO047084C4] Blanckenhorn W. U. (1998). Adaptive phenotypic plasticity in growth, development, and body size in the yellow dung fly. *Evolution (N. Y)* 52, 1394-1407. 10.1111/j.1558-5646.1998.tb02021.x 28565396

[BIO047084C5] Blueweiss A. L., Fox H., Kudzma V., Nakashima D., Peters R., Sams S. and Url S. (2013). Relationships between body size and some life history parameters. *Oecologia* 37, 257-272. 10.1007/BF00344996 28309655

[BIO047084C6] Chakraborty R., Vepuri V., Mhatre S. D., Paddock B. E., Miller S., Michelson S. J., Delvadia R., Desai A., Vinokur M., Melicharek D. J. et al. (2011). Characterization of a *Drosophila* alzheimer's disease model: pharmacological rescue of cognitive defects. *PLoS ONE* 6, e20799 10.1371/journal.pone.0020799 21673973 PMC3108982

[BIO047084C7] Chechi T. S., Ali Syed Z. and Prasad N. G. (2017). Virility does not imply immensity: Testis size, accessory gland size and ejaculate depletion pattern do not evolve in response to experimental manipulation of sex ratio in *Drosophila melanogaster*. *J. Insect Physiol.* 98, 67-73. 10.1016/j.jinsphys.2016.11.012 27913151

[BIO047084C8] Chen C., Jack J. and Garofalo R. S. (1996). The *Drosophila* insulin receptor is required for normal growth. *Endocrinology* 137, 846-856. 10.1210/endo.137.3.8603594 8603594

[BIO047084C9] Chinathamby K., Reina R. D., Bailey P. C. E. and Lees B. K. (2006). Effects of salinity on the survival, growth and development of tadpoles of the brown tree frog, *Litoria ewingii*. *Aust. J. Zool.* 54, 97-105. 10.1071/ZO06006

[BIO047084C10] Clark T. M., Flis B. J. and Remold S. K. (2004). Differences in the effects of salinity on larval growth and developmental programs of a freshwater and a euryhaline mosquito species (Insecta: Diptera, Culicidae). *J. Exp. Biol.* 207, 2289-2295. 10.1242/jeb.01018 15159433

[BIO047084C11] Croghan P. C. and Lockwood A. P. M. (1959). The composition of the haemolymph of the larva of *Drosophila melanogaster*. *J. Exp. Biol.* 37, 339-343.

[BIO047084C12] Danielsen E. T., Moller M. E. and Rewitz K. F. (2013). Nutrient signaling and developmental timing of maturation. *Curr. Top. Dev. Biol.* 105, 37-67. 10.1016/B978-0-12-396968-2.00002-6 23962838

[BIO047084C13] Das D. and Arur S. (2017). Conserved insulin signaling in the regulation of oocyte growth, development, and maturation. *Mol. Reprod. Dev.* 84, 444-459. 10.1002/mrd.22806 28379636 PMC5477485

[BIO047084C14] Davidowitz G. and Nijhout H. F. (2004). The physiological basis of reaction norms: the interaction among growth rate, the duration of growth and body size. *Integr. Comp. Biol.* 44, 443-449. 10.1093/icb/44.6.443 21676730

[BIO047084C15] Davidowitz G., Roff D. A. and Nijhout H. F. (2005). A physiological perspective on the response of body size and development time to simultaneous directional selection. *Integr. Comp. Biol.* 45, 525-531. 10.1093/icb/45.3.525 21676797

[BIO047084C16] De Jong G. and Bochdanovits Z. (2003). Latitudinal clines in Drosophila melanogaster: body size, allozyme frequencies, inversion frequencies, and the insulin-signalling pathway. *J. Genet.* 82, 207-223. 10.1007/BF02715819 15133196

[BIO047084C17] De Moed G. H., Kruitwagen C. L. J. J., De Jong G. and Scharloo W. (1999). Critical weight for the induction of pupariation in *Drosophila melanogaster*: genetic and environmental variation. *J. Evol. Biol.* 12, 852-858. 10.1046/j.1420-9101.1999.00103.x

[BIO047084C18] Diaz-Fleischer F., Arredondo J., Lasa R., Bonilla C., Debernardi D., Perez-Staples D. and Williams T. (2019). Sickly sweet: insecticidal polyols induce lethal regurgitation in dipteran pests. *Insects* 10, 53 10.3390/insects10020053 PMC6410098 30759873

[BIO047084C19] Elhassan E. and Schrier R. W. (2015). Disorders of Extracellular Volume. In *Comprehensive Clinical Nephrology* (ed. Johnson R. J., Feehally J. and Floege J.), pp. 85-99. Philadelphia, PA: Saunders.

[BIO047084C20] Fiocca K., Barrett M., Waddell E. A., McNair C., O'Donnell S. and Marenda D. R. (2019). Mannitol ingestion causes concentration-dependent, sex-specific mortality in adults of the fruit fly (*Drosophila melanogaster*). *PLoS ONE* 14, e0213760 10.1371/journal.pone.0213760 31150400 PMC6544200

[BIO047084C21] GraphPad Software, Inc. (2018). GraphPad Prism for Windows, Version 8.0.0. La Jolla: GraphPad Software.

[BIO047084C22] Grembecka M. (2015). Sugar alcohols—their role in the modern world of sweeteners: a review. *Eur. Food Res. Technol.* 241, 1-14. 10.1007/s00217-015-2437-7

[BIO047084C23] Hu J. S., Gelman D. B., Salvucci M. E., Chen Y. P. and Blackburn M. B. (2010). Insecticidal activity of some reducing sugars against the sweet potato whitefly, *Bemisia tabaci*, Biotype B. *J. Insect Sci.* 10, 203 10.1673/031.010.20301 21268696 PMC3029359

[BIO047084C24] Huang X.-L., Lan X., He H.-M. and Xue F.-S. (2018). Effect of rearing conditions on the correlation between larval development time and pupal weight of the rice stem borer, *Chilo suppressalis*. *Ecol. Evol.* 8, 12694-12701. 10.1002/ece3.4697 30619574 PMC6308898

[BIO047084C25] IBM Corp. (2016). IBM SPSS Statistics for Windows, Version 24.0. Armonk, IBM Corp.

[BIO047084C26] Jamieson P. R., Le A. S. and Mulderrig K. B. (2001). Sorbitol and mannitol. In *Alternative Sweetners* (ed. Nabors L. O. and Gelardi R. C.), pp. 333-348. New York, NY: Marcel Dekker, Inc.

[BIO047084C27] Kikuta S. (2018). Response of *Tribolium castaneum* to dietary mannitol, with remarks on its possible nutritive effects. *PLoS ONE* 13, e0207497 10.1371/journal.pone.0207497 30427916 PMC6235386

[BIO047084C28] Kim J. and Neufeld T. P. (2015). Dietary sugar promotes systemic TOR activation in *Drosophila* through AKH-dependent selective secretion of Dilp3. *Nat. Commun.* 6, 6846 10.1038/ncomms7846 25882208 PMC4402654

[BIO047084C29] Lewis D. H. and Smith D. C. (1967). Sugar alcohols (Polyols) in fungi and green plants. I. Distribution, physiology and metabolism. *New Phytol.* 66, 143-184. 10.1111/j.1469-8137.1967.tb05997.x

[BIO047084C30] Lihoreau M., Poissonnier L.-A., Isabel G. and Dussutour A. (2016). *Drosophila* females trade off good nutrition with high-quality oviposition sites when choosing foods. *J. Exp. Biol.* 219, 2514-2524. 10.1242/jeb.142257 27284071

[BIO047084C31] Lin X., Yao Y., Wang B., Emlen D. J. and Lavine L. C. (2016). Ecological trade-offs between migration and reproduction are mediated by the nutrition-sensitive insulin-signaling pathway. *Int. J. Biol. Sci.* 12, 607-616. 10.7150/ijbs.14802 27143957 PMC4852207

[BIO047084C32] Lu H.-L. and Pietrantonio P. V. (2011). Insect insulin receptors: Insights from sequence and caste expression analyses of two cloned hymenopteran insulin receptor cDNAs from the fire ant. *Insect Mol. Biol.* 20, 637-649. 10.1111/j.1365-2583.2011.01094.x 21797944

[BIO047084C33] Makinen K. K. and Soderling E. (1980). A quantitative study of mannitol, sorbitol, xylitol, and xylose in wild berries and commercial fruits. *J. Food Sci.* 45, 367-371. 10.1111/j.1365-2621.1980.tb02616.x

[BIO047084C34] Matthews B. E. (1985). The influence of temperature and osmotic stress on the development and eclosion of hookworm eggs. *J. Helminthol.* 59, 217-224. 10.1017/S0022149X00007975 3934257

[BIO047084C35] Matzkin L. M., Johnson S., Paight C., Bozinovic G. and Markow T. A. (2011). Dietary protein and sugar differentially affect development and metabolic pools in ecologically diverse *Drosophila*. *J. Nutr.* 141, 1127-1133. 10.3945/jn.111.138438 21525254

[BIO047084C36] Mirth C. K. and Riddiford L. M. (2007). Size assessment and growth control: how adult size is determined in insects. *BioEssays* 29, 344-355. 10.1002/bies.20552 17373657

[BIO047084C37] Mirth C. K. and Shingleton A. W. (2012). Integrating body and organ size in *Drosophila*: recent advances and outstanding problems. *Front. Endocrinol. (Lausanne)* 3, 49 10.3389/fendo.2012.00049 22654869 PMC3356080

[BIO047084C38] Morris S. N. S., Coogan C., Chamseddin K., Fernandez-Kim S. O., Kolli S., Keller J. N. and Bauer J. H. (2012). Development of diet-induced insulin resistance in adult *Drosophila melanogaster*. *Biochim. Biophys. Acta* 1822, 1230-1237. 10.1016/j.bbadis.2012.04.012 22542511 PMC3601833

[BIO047084C39] Musselman L. P., Fink J. L., Narzinski K., Ramachandran P. V., Hathiramani S. S., Cagan R. L. and Baranski T. J. (2011). A high-sugar diet produces obesity and insulin resistance in wild-type *Drosophila*. *Dis. Model. Mech.* 4, 842-849. 10.1242/dmm.007948 21719444 PMC3209653

[BIO047084C40] Nicolson S. W. (1994). Eucalyptus nectar: production, availability, composition and osmotic consequences for the larva of the eucalypt nectar fly, *Drosophila flavohirta*. *Suid-Afrikaanse Tydskr. vir Wet.* 90, 75-79.

[BIO047084C41] Niewalda T., Singhal N., Fiala A., Saumweber T., Wegener S. and Gerber B. (2008). Salt processing in larval *Drosophila*: choice, feeding, and learning shift from appetitive to aversive in a concentration-dependent way. *Chem. Senses* 33, 685-692. 10.1093/chemse/bjn037 18640967 PMC2565773

[BIO047084C42] Nijhout H. F., Roff D. A. and Davidowitz G. (2010). Conflicting processes in the evolution of body size and development time. *Philos. Trans. R. Soc. B Biol. Sci.* 365, 567-575. 10.1098/rstb.2009.0249 PMC2817141 20083633

[BIO047084C43] O'Donnell S., Baudier K., Fiocca K. and Marenda D. R. (2017). Erythritol ingestion impairs adult reproduction and causes larval mortality in *Drosophila melanogaster* fruit flies (Diptera: Drosophilidae). *J. Appl. Entomol.* 00, 1-6. 10.1111/jen.12409

[BIO047084C44] Onishi H. and Suzuki T. (1968). Production of D-mannitol and glycerol by yeasts. *Appl. Microbiol.* 16, 1847-1952. 5749751 10.1128/am.16.12.1847-1852.1968 PMC547782

[BIO047084C45] Ormerod K. G., LePine O. K., Abbineni P. S., Bridgeman J. M., Coorssen J. R., Mercier A. J. and Tattersall G. J. (2017). *Drosophila* development physiology, behavior, and lifespan are influenced by altered dietary composition. *Fly (Austin)* 11, 153-170. 10.1080/19336934.2017.1304331 28277941 PMC5552271

[BIO047084C46] Reis T. (2016). Effects of synthetic diets enriched in specific nutrients on *Drosophila* development, body fat, and lifespan. *PLoS ONE* 11, e0146758 10.1371/journal.pone.0146758 26741692 PMC4704830

[BIO047084C47] Roff D. A. (2000). Trade-offs between growth and reproduction: an analysis of the quantitative genetic evidence. *Jounal Evol. Biol.* 13, 434-445. 10.1046/j.1420-9101.2000.00186.x

[BIO047084C48] Rovenko B. M., Kubrak O. I., Gospodaryov D. V., Perkhulyn N. V., Yurkevych I. S., Sanz A., Lushchak O. V. and Lushchak V. I. (2015). High sucrose consumption promotes obesity whereas its low consumption induces oxidative stress in *Drosophila melanogaster*. *J. Insect Physiol.* 79, 42-54. 10.1016/j.jinsphys.2015.05.007 26050918

[BIO047084C49] Saha B. C. and Racine F. M. (2011). Biotechnological production of mannitol and its applications. *Appl. Microbiol. Biotechnol.* 89, 879-891. 10.1007/s00253-010-2979-3 21063702

[BIO047084C50] Santos M., Fowler K. and Partridge L. (1994). Gene-environment interaction for body size and larval density in *Drosophila melanogaster*: an investigation of effects on development time, thorax length and adult sex ratio. *Heredity (Edinb)* 72, 515-521. 10.1038/hdy.1994.69 8014062

[BIO047084C51] Schneider C. A., Rasband W. S. and Eliceiri K. W. (2012). NIH Image to ImageJ: 25 years of image analysis. *Nat. Methods* 9, 671-675. 10.1038/nmeth.2089 22930834 PMC5554542

[BIO047084C52] Systat Software, Inc. (2013). SigmaPlot for Windows, Version 12.5. San Jose: Systat Software.

[BIO047084C53] Takada T., Sato R. and Kikuta S. (2017). A mannitol/sorbitol receptor stimulates dietary intake in *Tribolium castaneum*. *PLoS ONE* 12, e0186420 10.1371/journal.pone.0186420 29023543 PMC5638539

[BIO047084C54] Thomas R. H. (1993). Ecology of body size in *Drosophila buzzatii*: untangling the effects of temperature and nutrition. *Ecol. Entomol.* 18, 84-90. 10.1111/j.1365-2311.1993.tb01084.x

[BIO047084C55] Tyler M. S. (2000). Development of the Fruit Fly: *Drosophila melanogaster*. In *Developmental Biology: A Guide for Experimental Study*, pp. 85-106. Sunderland, MA: Sinauer Associates Inc. Publishers.

[BIO047084C56] Wheeler D. E., Buck N. and Evans J. D. (2006). Expression of insulin pathway genes during the period of caste determination in the honey bee, *Apis mellifera*. *Insect Mol. Biol.* 15, 597-602. 10.1111/j.1365-2583.2006.00681.x 17069635 PMC1761130

[BIO047084C57] Wu Q. and Brown M. R. (2006). Signaling and function of insulin-like peptides in insects. *Annu. Rev. Entomol.* 51, 1-24. 10.1146/annurev.ento.51.110104.151011 16332201

[BIO047084C58] Wu C.-S., Gomez-Mestre I. and Kam Y.-C. (2012). Irreversibility of a bad start: early exposure to osmotic stress limits growth and adaptive developmental plasticity. *Oecologia* 169, 15-22. 10.1007/s00442-011-2170-2 22037992

[BIO047084C59] Xiao H. J., Xue F. S., Liu Y. Q. and Zhu X. F. (2005). Comparison of photoperiodic response between rice- and water-oat populations of *Chilo suppressalis* (Walker). *Acta Entornologica Sin.* 48, 749-753.

[BIO047084C60] Xu H.-J., Xue J., Lu B., Zhang X.-C., Zhuo J.-C., He S.-F., Ma X.-F., Jiang Y.-Q., Fan H.-W., Xu J.-Y. et al. (2015). Two insulin receptors determine alternative wing morphs in planthoppers. *Nature* 519, 464-467. 10.1038/nature14286 25799997

[BIO047084C61] Yao C. K., Tan H.-L., van Langenberg D. R., Barrett J. S., Rose R., Liels K., Gibson P. R. and Muir J. G. (2014). Dietary sorbitol and mannitol: food content and distinct absorption patterns between healthy individuals and patients with irritable bowel syndrome. *J. Hum. Nutr. Diet.* 27, 263-275. 10.1111/jhn.12144 23909813

[BIO047084C62] Zwaan B. J., Bijlsma R. and Hoekstra R. F. (1992). On the developmental theory of ageing. II. the effect of developmental temperature on longevity in relation to adult body size in *D. melanogaster*. *Heredity (Edinb)* 68, 123-130. 10.1038/hdy.1992.19 1548140

