# Supplementary material for: Larval mannitol diets increase mortality, prolong development and decrease adult body sizes in fruit flies (Drosophila melanogaster)
Source: Biol Open. 2020 Jan 2;8(12):bio047084. doi: 10.1242/bio.047084 (PMC6955208; doi:10.1242/bio.047084)
Supplement: Supplementary information [file biolopen-8-047084-s1.pdf]

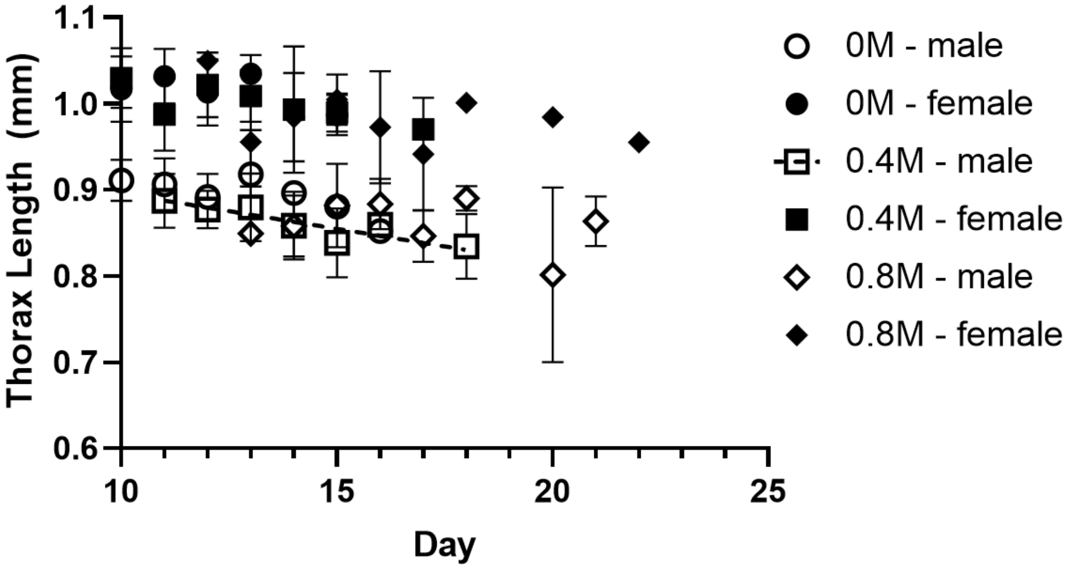

Fig S1. Effect of emergence day on adult thorax length. Linear regressions showing effect of emergence day on thorax length in males and females at each concentration. Only 0.4M-males saw a significant decreasing in thorax length as eclosion was more delayed (y=-0.0029x+0.9777; F=10.51, p=0.002, R^2=0.1534); slopes were not significantly different from one another (F=0.5298, p=0.7537). Error bars represent one standard deviation.

Biology Open • Supplementary information

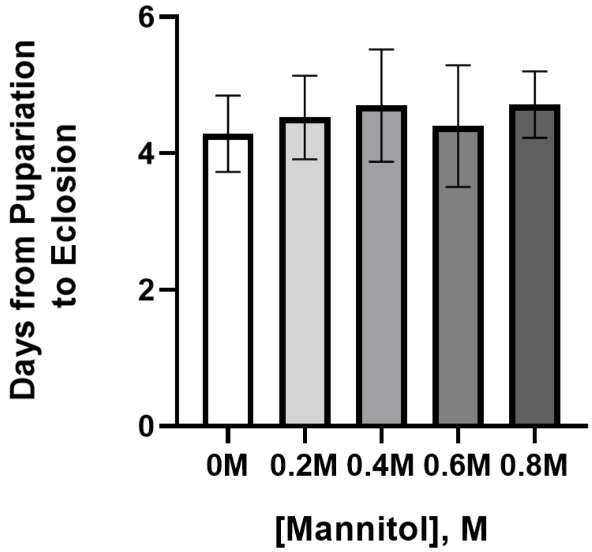

Fig S2. Time from pupariation to eclosion when ingesting increasing concentrations of mannitol. Time from pupariation to eclosion did not differ between control and any mannitol treatments (ANOVA, F=1.04, p=0.39). Error bars represent one standard deviation.

Biology Open • Supplementary information

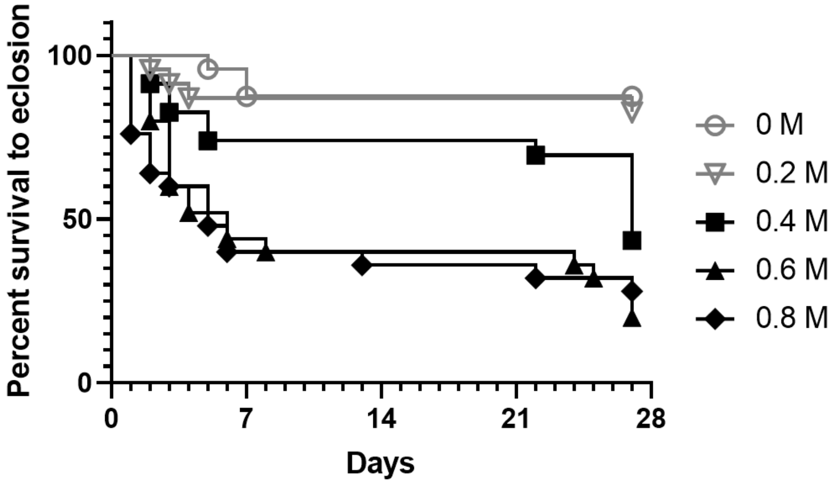

Fig S3. Percent survival of *D. melanogaster* larvae fed increasing concentrations of mannitol. Survival plots showing percent survival to eclosion versus post-hatching fly age given control food or foods with increasing concentrations of mannitol (0.2M to 0.8M). Observations were terminated at 27 days after egg laying (n=30eggs/treatment). Highly significant differences ($p<0.01$) from the control are in black, non-significant differences are in grey.
